# Comparative genomics uncovers organellar genome structural divergence in Caryophyllales and reveals widespread non-coding transcription in *Bougainvillea glabra* organellar

**DOI:** 10.1186/s12864-025-11891-5

**Published:** 2025-08-04

**Authors:** Shuo Zhang, Shengxin Chang, Xinge Lin, Shisong Xu, Qingyun Leng, Haiyan Li, Hernán Ariel López, Junmei Yin, Zhiqiang Wu, Junhai Niu

**Affiliations:** 1 National Key Laboratory for Tropical Crop Breeding/Key Laboratory of Gene Resources and Germplasm Enhancement in Southern China, MARA/Laboratory of Tropical Crops Germplasm Resources Genetic Improvement and Innovation of Hainan Province/Tropical Crops Genetic Resources Institute, Chinese Academy of Tropical Agricultural Sciences (CATAS), Haikou, Hainan Province 571101 China; 2 Shenzhen Branch, Guangdong Laboratory of Lingnan Modern Agriculture, Key Laboratory of Synthetic Biology, Ministry of Agriculture and Rural Affairs, Agricultural Genomics Institute at Shenzhen, Chinese Academy of Agricultural Sciences, Shenzhen, China; 3 State Key Laboratory of Tropical Crop Breeding, Shenzhen Branch, Laboratory of Lingnan Modern Agriculture, Key Laboratory of Synthetic Biology, Ministry of Agriculture and Rural Affairs, Agricultural Genomics Institute at Shenzhen, Chinese Academy of Agricultural Sciences, Shenzhen, Guangdong China; 4 Shaoguan University, Shaoguan, Guangdong Province 512005 China; 5 Multidisciplinary Workshop on Vascular Plants, Border Ecology Laboratory, University of Flores, Sede Comahue (UFLO), Rio Negro, Argentina; 6 The Botanical Garden of the Plottier City, Neuquen, Argentina; 7 Sanya Research Academy, Chinese Academy of Tropical Agriculture Sciences, Sanya , Hainan Province 572025 China

**Keywords:** Mitochondrion, Plastid, RNA editing, Caryophyllales, *Bougainvillea*

## Abstract

Plant organellar genomes play a crucial role in metabolism and adaptation. In this study, the organellar genomes and transcriptome of *Bougainvillea glabra* (Nyctaginaceae) were sequenced and assembled using PacBio sequencing and strand-specific RNA sequencing, respectively. Structural and evolutionary comparisons of the plastidial and mitochondrial genomes (plastome and mitogenome) were conducted among *Bougainvillea* and five other taxa within Caryophyllales to elucidate the similarities and divergences between these two organellar genomes at a detailed level. The plastome of *B. glabra* was assembled into a 154.7 kb circular molecule with a typical quadripartite structure, while the mitogenome was assembled into three stable circular molecules measuring 160.7 kb, 97.6 kb, and 64.3 kb, respectively. Reconstruction of the organellar transcripts revealed extensive transcriptional activity in the non-coding regions of the organellar genomes. However, the transcriptional activity and editing proportions of novel transcripts identified in these regions were significantly lower compared to those of conserved organellar transcripts. A tenfold difference in the number of RNA editing sites was observed between plastidial and mitochondrial transcripts (43 vs. 453), with the majority (70%) of these sites located at nonsilent sites within coding regions, exhibiting high editing efficiency (> 70%). The turnover rate of Caryophyllales mitogenomes was found to be, on average, 6.1 times faster than that of plastomes. In contrast, the nucleotide substitution rate in protein-coding genes was significantly higher in plastomes than in mitogenomes. Moreover, nonsilent nucleotide substitutions in genes encoding components of the electron transfer chain were more constrained compared to those in ribosomal protein-coding genes in both plastidial and mitochondrial genomes of Caryophyllales. Together, these findings provide vital genetic resources that enhance our understanding of the dynamic evolution and phylogenetic relationships within *Bougainvillea* and the broader Caryophyllales order.

**Supplementary Information:**

The online version contains supplementary material available at 10.1186/s12864-025-11891-5.

## Highlights

The *Bougainvillea glabra* mitogenome comprises three circular chromosomes with lengths of 160.7 kb, 97.6 kb, and 54.3 kb, resulting in a total genome size of 322.7 kb. The plastome was assembled as a 154.7 kb circular molecule with a typical quadripartite structure.

Analysis of organellar transcripts revealed widespread transcriptional activity in non-coding regions; however, the transcriptional activity and editing proportion of novel transcripts in these regions were significantly lower than those of conserved organellar transcripts.

A tenfold difference in the number of RNA editing sites was observed between plastidial and mitochondrial transcripts (43 vs. 453), with the majority (70%) of these sites located at nonsilent sites in coding regions, typically exhibiting high editing efficiency (> 70%).

The turnover rate of Caryophyllales mitogenomes was found to be, on average, 6.1 times higher than that of plastomes. In contrast, the nucleotide substitution rate in protein-coding genes was significantly higher in plastomes compared to mitogenomes. Furthermore, nonsilent nucleotide substitutions in genes encoding components of the electron transport chain were more constrained than those in ribosomal protein-coding genes in both plastidial and mitochondrial genomes of Caryophyllales.

**Supplementary Information:**

The online version contains supplementary material available at 10.1186/s12864-025-11891-5.

## Introduction

Plastids and mitochondria are two types of endosymbiotic organelles in plant cells, originating from the ancient uptake of cyanobacteria and α-Proteobacteria by ancestral host cells [1]. Unlike nuclear genomes, organellar genomes are mostly maternally inherited, making them valuable for studies in plant evolution, population genetics, and biotechnology [2]. However, obtaining intact organellar genomes from higher plants has long posed significant challenges. Conventional methods require complex organelle isolation and purification procedures prior to organellar DNA extraction to minimize nuclear DNA contamination—primarily resulting from the frequent transfer of genetic material from organellar genomes to the nuclear genome (i.e., nuclear mitochondrial transferred fragments, NUMTs) [3]. The process of organellar extraction is fraught with uncertainties due to the difficulty of determining organellar density, the fragility of organellar membranes in vitro, and the stringent ultracentrifugation conditions required [4]. As of April 2025, only 688 mitochondrial genomes and 12,989 plastid genomes of land plants had been deposited in the NCBI Genome database (https://www.ncbi.nlm.nih.gov/datasets/organelle/), compared to 3,517 plant nuclear genomes, which are typically thousands of times larger than organellar genomes. In recent years, advancements in long-read sequencing technologies have driven a transformative revolution in plant genome research [3] The longer sequencing reads offer distinct advantages for distinguishing DNA from different origins and assembling structurally complex genomes, enabling the direct assembly of plant organellar genomes from whole-genome sequencing data [5]. This approach has been successfully applied to assemble the organellar genomes of *Chenopodium quinoa* and *Camellia assamica* with the aid of reference genomes from closely related species [6, 7].

Caryophyllales is a diverse order of angiosperms found on every continent, with representatives inhabiting a wide range of terrestrial and aquatic environments. This order is divided into core Caryophyllales and non-core Caryophyllales, with the core group representing the primary lineage, predominantly composed of betalain-producing taxa [8]. Several studies have explored the evolutionary processes of Caryophyllales based on the sequence divergence of various organellar genes, complete plastomes, or mitogenomes [9–12]. However, comparative evolutionary analyses involving both organellar genomes in Caryophyllales have been limited, largely due to the lack of comprehensive organellar genome data until recent years. To date, organellar genome sequences have been assembled with high fidelity and released for six species within the order Caryophyllales. Five of these belong to the core Caryophyllales (*Beta vulgaris*, *Chenopodium quinoa*, *Spinacia oleracea*, *Silene latifolia*, and *B. glabra*), while one (*Nepenthes ventricosa*) represents the non-core group. Among these, the organellar genomes of *B. glabra* (*Nyctaginaceae*), a widely cultivated ornamental plant in tropical and subtropical regions, are presented in this study. By mining organellar DNA fragments from a high-coverage PacBio sequencing dataset (100×) generated in-house, the organellar genomes were assembled. Illumina strand-specific RNA sequencing was simultaneously performed to investigate organellar transcripts and RNA editing sites. The structural organization and gene content of the *B. glabra* organellar genomes were compared with those of the five other Caryophyllales taxa. Additionally, mutation rate analyses were conducted across these taxa to provide insights into their evolutionary dynamics.

## Results

### Organellar genome features

**Fig. 1:**
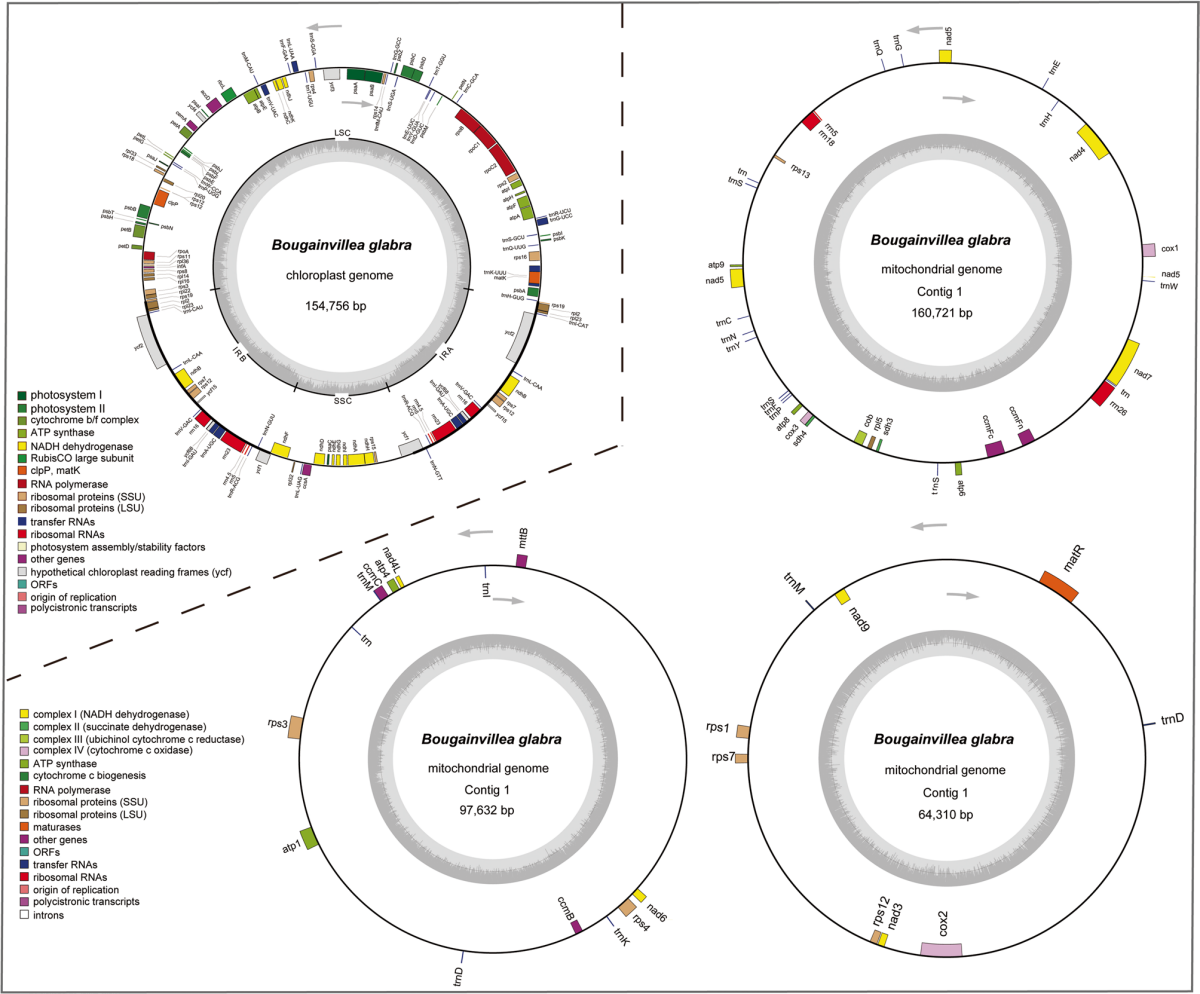
Schematic of the organellar genomes of *B. glabra* drawn by Organellar GenomeDRAW 1.3.1

*B*. *glabra* plastome was assembled into a 154.7 kb single circular contig (GenBank accession number: MN432176) with 7781 × PacBio sequencing depth, while its mitogenome was assembled into three circular contigs with sizes of 160.7 kb, 97.6 kb and 64.3 kb (GenBank accession numbers: MN432177, MN432178 and MN432175), respectively, with 2620 × sequencing depth on average (Fig. 1 and S1). The plastome has an overall GC content of 36.4%, which is 7.7% lower than that of the mitogenome, and a similar difference in GC content between plastome and mitogenome was also found in other Caryophyllales taxa (Table 1). Only a typical 25.4 kb inverted repeat is shared in *B*. *glabra* plastome, and no large repeats (≥ 1 kb) were found in *B*. *glabra* mitogenome.

**Table 1:** The organellar features of six Caryophyllales taxa

Category	*Nepenthes *	*Bougainvillea*	*Silene *	*Beta *	*Chenopodium *	*Spinacia *	CV
Size (kb)	156.4/520.8	154.8/322.7	151.7/253.4	149.7/365.0	152.1/315.0	150.7/329.6	0.015/0.27
GC%	37.1/44.2	36.4/44.1	36./42.6	37.0/43.9	37.2/43.8	36.8/43.4	0.0090/0.014
Content of large repeats ≥ 1 kb	25.2/21.1	25.4/0	25.9/10.5	24.4/29.1	25.2/5.3	25.1/7.3	0.017/0.85
Content of repeats < 1 kb	2.6/40.1	2.9/20.6	2.6/18.1	3.1/36.5	3.1/29.6	2.8/51.7	0.073/0.35
Content of tandem repeats ≥ 7 bp (kb)	0.89/0.71	1.23/1.18	0.59/1.74	0.73/1.56	0.71/0.57	0.18/1.06	0.44/0.37
Content of tandem repeats < 7 bp (kb)	1.98/4.02	1.86/2.99	1.76/1.97	1.72/2.61	1.38/2.19	1.63/2.05	0.11/0.27
Total gene number	111/58	114/55	111/40	112/54	112/52	112/52	0.0090/0.11
Number of protein-coding genes	77/37	80/33	77/28	78/30	78/30	78/30	0.013/0.093
rRNA gene number	4/3	4/3	4/3	4/3	4/3	4/3	0/0
tRNA gene number	30/18	30/19	30/9	30/21	30/19	30/19	0/0.22
Gene density (/kb)	0.85/0.12	0.88/0.17	0.88/0.16	0.89/0.16	0.88/0.17	0.89/0.16	0.018/0.12
MTPTs content (kb)	29.5	2.9	1.9	8.7	6.0	7.5	0.99

**Fig. 2:**
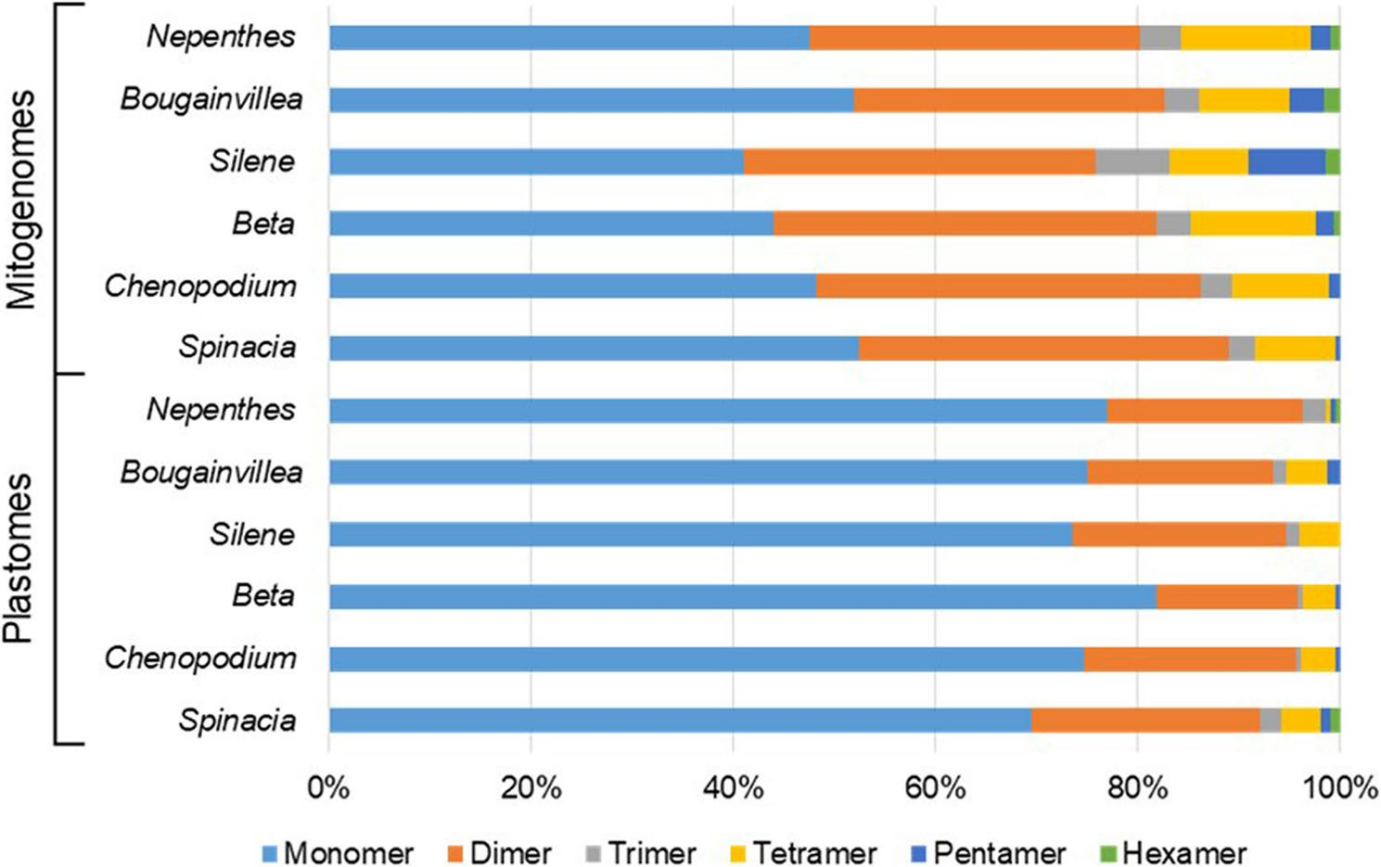
The motifs statistics of tandem repeats of Caryophyllales organellar genomes

To assess the structural stability of the *B*. *glabra* mitogenome, repeat sequences longer than 50 bp, along with their flanking regions (± 200 bp), were subjected to BLASTN analysis against the organellar PacBio reads. The results revealed that all matched hits exhibited a minimum coverage of 85%. A manual examination of hits with relatively low coverage (85–90%) found no evidence of repeat-mediated recombination. The observed imperfect matches were attributed to the inherent limitations in sequencing accuracy of the PacBio technology. A total of 235 tandem repeats (TRs) were identified in the *B*. *glabra* plastome using MISA, with the majority classified as monomers (71.5%), followed by dimers (17.4%), trimers (1.3%), tetramers (3.8%), and pentamers (1.3%). In contrast, 332 TRs were detected in the *B*. *glabra* mitogenome, where the relative proportions of higher-order polymers were generally greater than those observed in the plastome, except for monomers (Fig. 2). This pattern was also evident in the organellar genomes of other Caryophyllales taxa (Fig. 2) and *C. assamica* [7], highlighting the greater conservation of plastomes compared to mitogenomes with respect to repeat features. Furthermore, comparisons of the coefficient of variation (CV = σ/µ) for genome parameters among Caryophyllales organellar genomes revealed that, apart from TR content, the variability in genome size, GC content, and repeat content was consistently lower in plastomes than in mitogenomes (Table 1).

**Fig. 3:**
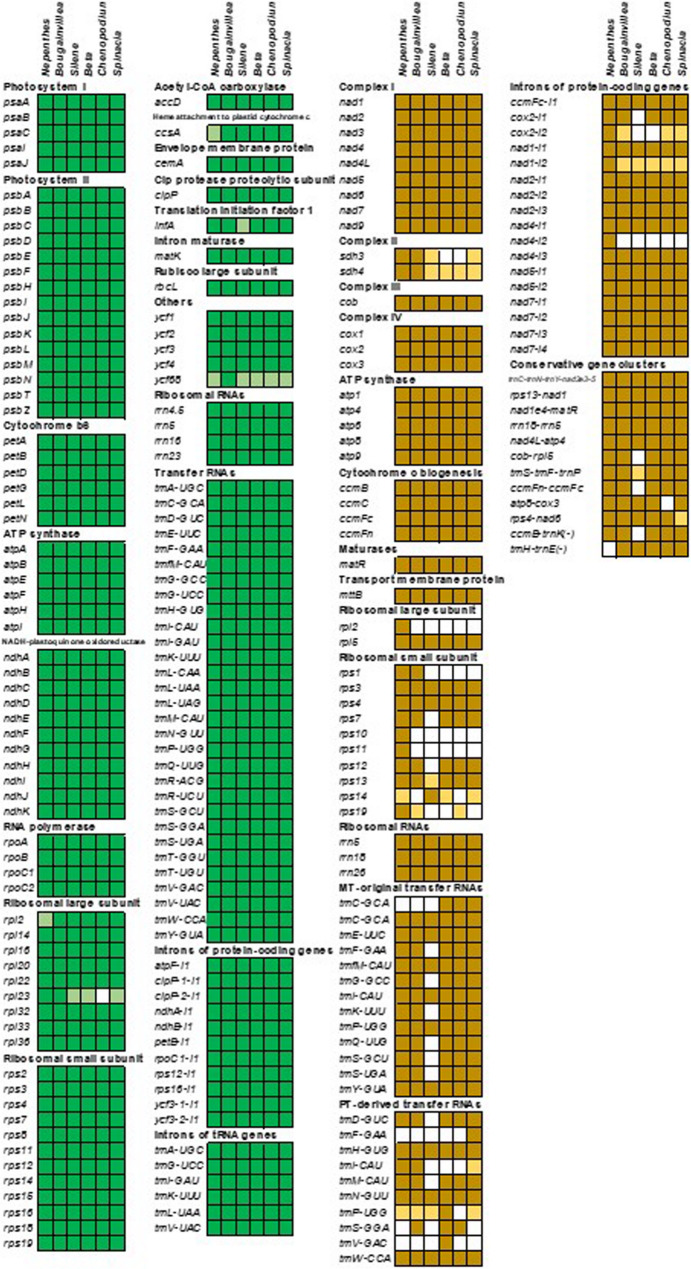
Organellar gene features among six Caryophyllales taxa. Boxes filled with dark and light colors denote true genes and pseudogenes, respectively. Blank boxes denote the lost genes. Green and yellow colors indicate plastidial and mitochondrial gene features, respectively

The gene content and density of the *B*. *glabra* plastome are substantially higher than those of its mitogenome, a trend consistent with observations in five other Caryophyllales taxa (Fig. 3; Table 1). Compared to other core Caryophyllales, the *B*. *glabra* organellar genomes exhibit abundant gene content, including the presence of the uncommon plastidial genes *rpl23* and *ycf68*, as well as the mitochondrial genes *rps1*, *sdh3*, *sdh4*, and *trnI-CAU* (Fig. 3). A total of 22 *B*. *glabra* organellar genes contain introns, six of which are plastidial tRNA genes, with the remainder being protein-coding genes (Fig. 3). All plastidial introns, along with the mitochondrial introns in *nad4*, *nad7*, *cox2*, and *ccmFc*, undergo trans-splicing, while the introns in mitochondrial *nad1*, *nad2*, and *nad5* are cis-spliced. These introns are also conserved in the organellar genomes of other core Caryophyllales taxa, except a *cox2* intron, which is absent in the genus *Silene*. In the mitogenome of the non-core Caryophyllales taxon (*Nepenthes*), three additional mitochondrial introns are present in the *cox2*, *nad1*, and *nad4* genes. These introns are also shared with other major taxa of seed plants and gymnosperms [13].

Seven mitochondrial tRNA genes in *B*. *glabra* were identified as plastid-derived, exhibiting high sequence similarity to plastidial DNA sequences (Fig. 3). Among these, *trnH* (GUG), *trnN* (GUU), and *trnW* (CCA) are shared across all Caryophyllales mitogenomes, indicating an ancient transfer event in the common ancestors of Caryophyllales. Four instances of gene overlap were observed in the organellar genomes of Bougainvillea and other Caryophyllales taxa, including the plastidial *psbD*-*psbC* linkage and the mitochondrial *cox3*-*sdh4*, *ccmC*-*trnM* (CAU, plastidial origin), and *rps3*-*rpl16* linkages. Among these, the plastidial *psbD* and *psbC* genes are known to exhibit translational coupling and are widely distributed in other flowering plants, algae, and cyanobacteria [14]. Two cases of gene insertion into tRNA genes were identified in the *B*. *glabra* plastome: *ycf68*-into-*trnI* (GAU) and *matK*-into-*trnK* (UUU). While relics of the *ycf68*-into-*trnI* insertion are present in other Caryophyllales plastomes due to the pseudogenization of *ycf68*, the *matK* insertion into *trnK* has been reported in species ranging from algae to angiosperms. This insertion is hypothesized to have first occurred in a primitive streptophyte following the divergence of chlorophytes and Mesostigma [15].

The coding sequence (CDS) lengths of organellar protein-coding genes in Caryophyllales are typically less than 1 kb (Fig. S2A), with only four plastidial CDSs exceeding 2.5 kb: *rpoB*, *rpoC2*, *ycf1*, and *ycf2*. The intron lengths of plastidial protein-coding and tRNA genes are significantly shorter than those of mitochondrial introns, as confirmed by a Mann-Whitney test (*P* < 0.05), with a boundary of approximately 1 kb (Fig. S2). The CV for intron lengths in organellar genomes is significantly higher than that for CDSs and tRNAs. Additionally, mitochondrial rRNA genes exhibit high variability in length, primarily due to substantial changes in the *rrn18* and *rrn26* genes (Fig. S3).

### Diversity of organellar structure and sequence in Caryophyllales

**Fig. 4:**
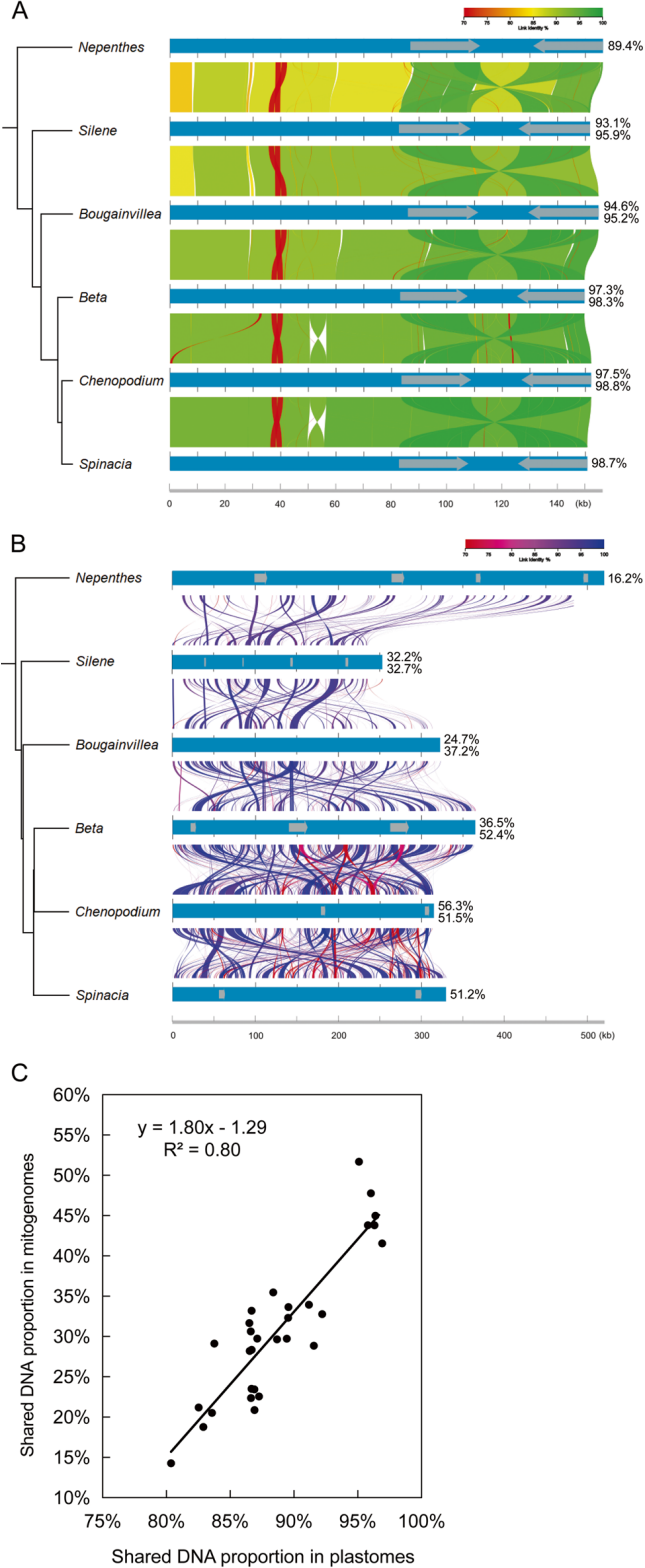
Shared organellar DNAs among six Caryophyllales taxa. Charts (**A**) and (**B**) display the synteny components of Caryophyllale plastomes and mitogenomes, respectively. Left trees give the similarity relationship of Caryophyllales organellar genomes using single cluster method on the matrix of the mean percentage of shared DNAs (Figure S3) after removing redundant large repeats. Right values give the percentage of shared DNAs. Gray blocks denote the locations of large repeats. **C** Scatter plot of proportions of shared DNAs of Caryophyllales plastomes and mitogenomes

The synteny analysis revealed a high degree of conservation in the plastid genomes across Caryophyllales species, with only a single 7-kb reverse rearrangement observed in the *Chenopodium quinoa* plastome compared to other Caryophyllales plastomes. In contrast, the mitogenomes of Caryophyllales exhibit highly fragmented structures, characterized by extensive rearrangements and inconsistent regions, even among closely related taxa such as *Chenopodium quinoa* and *Spinacia oleracea* (Fig. 4). According to the TimeTree website, the Caryophyllales order diverged approximately 85 million years ago (Mya). Over an evolutionary span of ~ 85 million years, approximately 90% of the syntenic regions are retained in Caryophyllales plastomes. However, during the divergence of *Beta vulgaris*, *Chenopodium quinoa*, and *Spinacia oleracea* mitogenomes (~ 37.4–43.3 Mya), only about 50% of syntenic DNA is preserved, decreasing further to ~ 30% when comparing these mitogenomes with those of *B*. *glabra*, *Silene latifolia*, and *Nepenthes ventricosa*, which diverged ~ 52.2–85 Mya (Fig. S4).

Despite the high rearrangement rates in Caryophyllales mitogenomes, 12 conserved gene clusters persist within syntenic regions. These include *trnC* (GCA)-*trnN* (GUU)-*trnY* (GUA)-*nad2e3-e5*, *rps13*-*nad1*, *nad1e4*-*matR*, *rrn18*-*rrn5*, *nad4L*-*atp4*, *cob*-*rpl5*, *trnS* (GCU)-*trnF* (GAA)-*trnP* (UUG), *ccmFn*-*ccmFc*, *atp8*-*cox3*, *rps4*-*nad6*, *ccmB*-*trnK* (UUU), and *trnH* (GTG)-*trnE* (UUC). Except for the *ccmB*-*trnK* and *trnH*-*trnE* clusters, which exhibit reverse gene orientations, all clusters are oriented uniformly (Fig. 3).

In Caryophyllales mitogenomes, the DNA turnover rate ranges from 0.7% to 1.5% (1.9–4.9 kb per million years), at least eight times higher than the corresponding rate in plastomes (Table S1). Furthermore, a positive correlation (R² = 0.8) was observed between the proportions of DNA shared between plastomes and mitogenomes (Fig. 4). Mitochondrial plastid transferred fragments (MTPTs) represent a significant component of mitogenome turnover, with their contribution ranging from 1.9 kb (0.8%) to 29.5 kb (5.9%) across Caryophyllales mitogenomes (Table 1). Notably, no exogenous mitochondrial DNA insertions were detected in Caryophyllales plastomes.

### Category of organellar transcripts of *Bougainvillea glabra*

The read distribution of the RNA-seq data derived from Bougainvillea leaf samples across organellar chromosomes exhibited the expected strand-specific characteristics (Fig. S5). The majority of transcriptomic reads (81.6%) were derived from the plastid genome, aligning with findings from nine other plastid-bearing species [16] (Table S2). Although the mitochondrial transcriptome accounted for a smaller proportion (7.3%) of the total sequencing library, it still provided sufficient coverage for comprehensive mitochondrial transcriptomic analysis. Notably, the sequencing depth for mitochondrial CDS regions exceeded 10× and 100× for 100% and 97.6% of the sites, respectively (Table S3). In fact, nearly all nucleotide sites in the *B*. *glabra* organellar genomes exhibited sequencing depths greater than 10×, with the plastome and mitogenome achieving coverage of 99.1% and 96.8%, respectively. Given that CDS regions represent only 48.7% of the plastome and 9.3% of the mitogenome, this extensive coverage strongly suggests that non-coding regions of the organellar genomes are actively transcribed.

After filtering transcripts with FPKM ≥ 0.5 and coverage ≥ 3, a significant number of novel organellar transcripts were identified. These were categorized into three types: intergenic region transcripts (u), intronic transcripts (i), and antisense transcripts of conserved CDSs (x). Thirty-three novel transcripts were identified in the plastid genome, all classified as x-type (Table S4 and Fig. S6). Four of these were predicted to be weakly coding RNAs (cRNAs) by CPC analysis: *BVpt_33* (960 bp), which crosses the antisense strand of the *ndhB* intron, is longer than previously known *ndhB* antisense transcripts (450–500 bp) identified in the plastomes of *Arabidopsis*, tobacco, and poplar [17]. Additionally, *BVpt_26* and *BVpt_27*, located on the antisense strand of the *rrn23* gene, encode peptides homologous to a metal transporter protein and an expansin protein, respectively (Table S4). Of the remaining 29 novel plastidial transcripts, 93% were classified as long non-coding RNAs (lncRNAs), characterized by open reading frames (ORFs) with lengths greater than 200 bp and fewer than 120 amino acids.

In the mitochondrial genome, 92 novel transcripts were identified, with a distribution density approximately 10% higher than that observed in the plastome. Ten of these mitochondrial transcripts were predicted as cRNAs, with coding lengths ranging from 36 to 303 bp (Table S4). Interestingly, three of the i-type cRNAs showed sequence homology (identity > 85%) with mitochondrial DNAs from distant species: *BVmt2_4* (192 bp) from *Hyoscyamus* and *Nicotiana* (Solanaceae), *BVmt2_21* (232 bp) from *Styphnolobium* (Fabaceae), *Boea*, and *Haberlea* (Lamiales), and *BVmt3_14* (342 bp) from *Populus*, with 54% coverage. These ORFs, however, lacked homologous sequences in other known Caryophyllales mitogenomes in NCBI-Genome and Ensembl (http://plants.ensembl.org). Seven mitochondrial cRNAs were categorized as x-type, corresponding to eight known mitochondrial genes (Table S4). A blastp-NR search revealed that the peptides encoded by *BVmt2_21* and *BVmt2_25* exhibited significant homology (identity > 85%, coverage > 65%) with retrovirus-related Pol polyproteins from transposons. Additionally, 82 ncRNAs were predicted in the mitochondrial genome, with 97.6% identified as lncRNAs, typically shorter than 2 kb (Table S4).

These results significantly expand our understanding of organellar transcriptomes in *B*. *glabra*, highlighting the transcriptional complexity and potential functional roles of non-coding regions, as well as providing new insights into the evolutionary dynamics of mitochondrial and plastidial genomes.

### RNA editing sites in organellargenome of *Bougainvillea glabra*

**Fig. 5:**
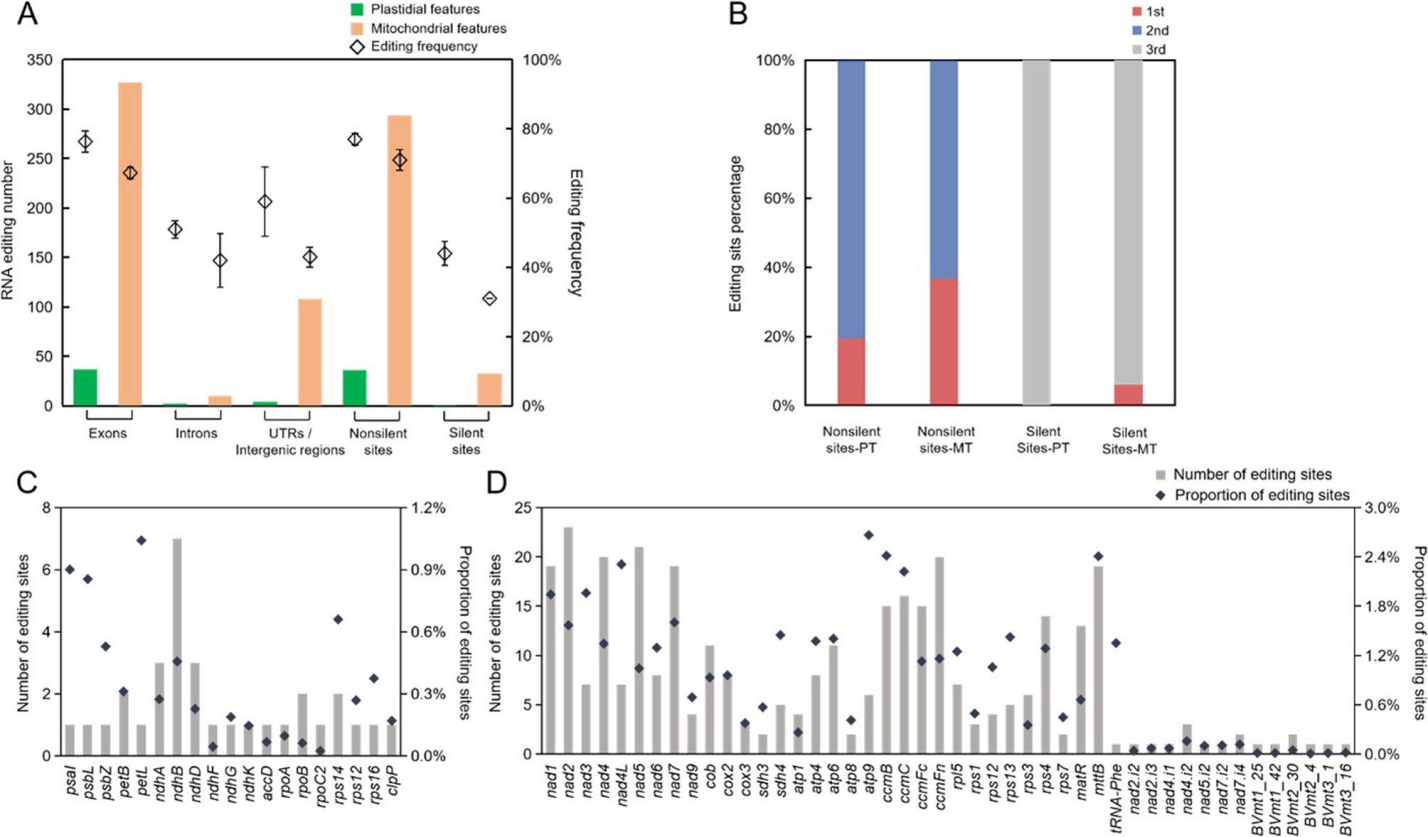
Number and frequency of RNA editing sites of different organellar features (**A**) and proportion of RNA editing at different codon sites (**B**). Error bars of editing frequency denote the standard errors. Number and proportion of RNA editing sites of CDSs, introns and novel transcripts of plastids (**C**) and mitochondria (**D**)

As a form of post-transcriptional modification, C-to-U RNA editing was identified at 43 sites in plastid transcripts and 453 sites in mitochondrial transcripts of *B. glabra*. These editing events were detected using Fisher’s exact test in REDItools, applied to each strand of the organellar chromosomes (Table S5). Notably, the majority of RNA editing events (> 70%) occurred at nonsilent sites within gene exons (Fig. 5A and B), a pattern consistent with observations in other land plant organelles [18–24]. These nonsilent editing events are widely regarded as RNA repair mechanisms, essential for maintaining normal plant development [25–27].

Analysis of editing frequencies revealed a significantly higher proportion of nonsilent editing compared to silent editing (*P* < 0.001), with nonsilent editing ratios averaging 77% vs. 44% in plastids and 71% vs. 31% in mitochondria (Fig. 5A). Among these, edits predominantly targeted the first and second codon positions, with the second position accounting for 80% and 63% of nonsilent editing events in plastids and mitochondria, respectively (Fig. 5A and B).

In plastids, RNA editing sites were detected in only 18 protein-coding genes (24%), whereas nearly all mitochondrial protein-coding genes exhibited editing, except for the *cox1* gene. The proportion of editing sites in plastidial genes was generally less than 0.3%, while in mitochondrial genes, it exceeded 1.0%. Additionally, six novel mitochondrial transcripts were identified as targets of RNA editing (Fig. 5C and D), the editing proportion of which was extremely lower than that of the CDSs of mitochondrial protein-coding genes but similar with that of the introns, which indicate their low purifying need on transcriptional level.

The codons for hydrophilic serine (S) and hydrophobic proline (P) were identified as the primary targets of RNA editing in both organelles (Fig. 5C and D). Of these, 89% of S codons and 66% of P codons were converted to hydrophobic leucine (L) codons in plastids and mitochondria, respectively. Notably, L codons were found to be the most abundant amino acid codons in *B. glabra* organellar protein-coding genes, irrespective of RNA editing, followed by S and P codons, which ranked as the second and third most abundant codons, respectively (Fig. S7).

### Substitution rate

**Fig. 6:**
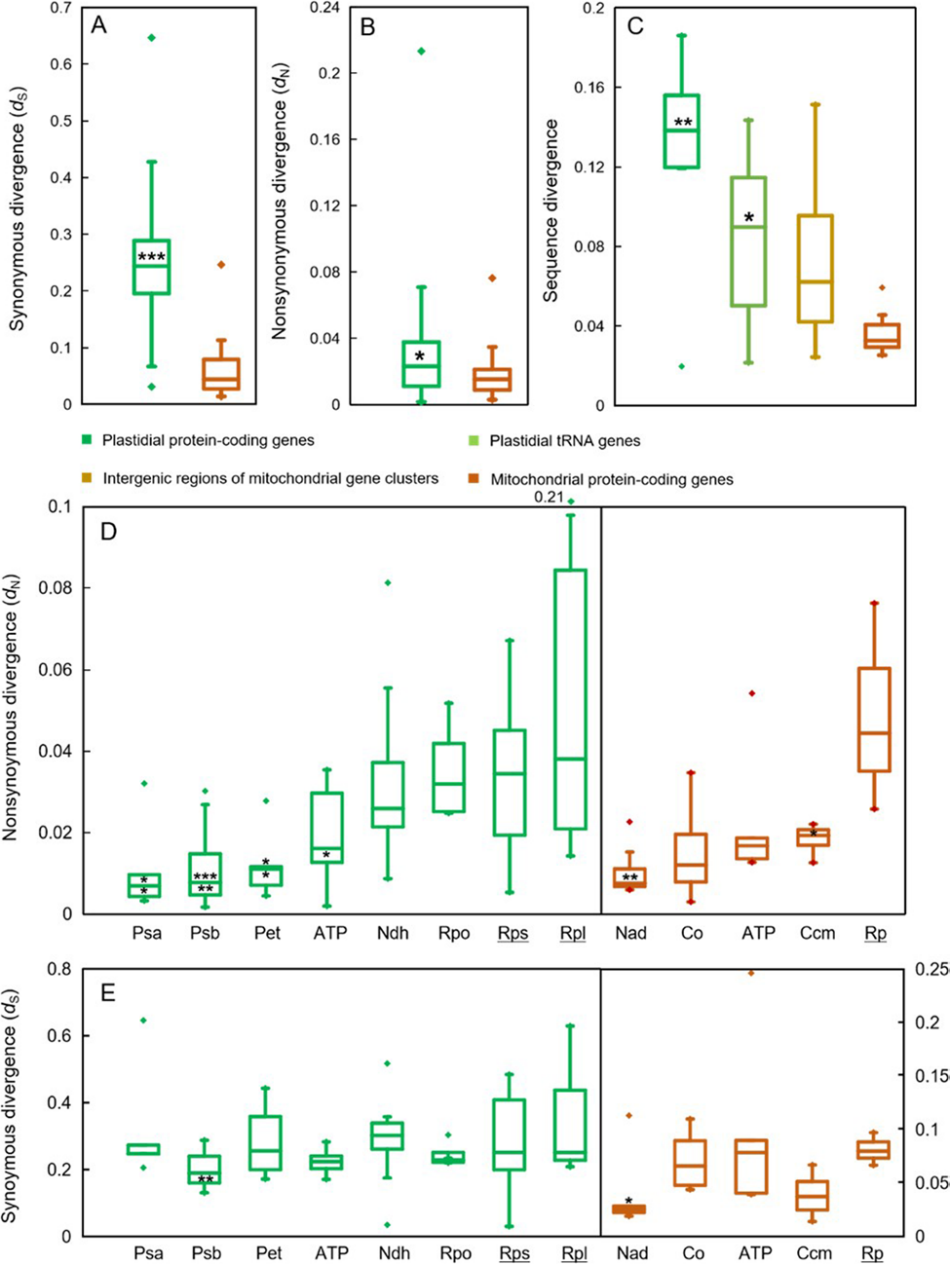
Synonymous (**A**) and nonsynonymous substitution rate (**B**) of organellar CDSs and the substitution rate of organellar introns and intergenic regions of gene clusters (**C**). Significance level for plastidial sequences is compared to corresponding mitochondrial sequences. *, ** and *** denotes a significant different with P less than 0.05, 0.01 and 0.001 under a T test. Boxes show the 25% and75% quartiles of the data points, and the horizontal lines within the boxes indicate the medians. Whisker lines encompass the range of all non-outlier data points. Points outside the whisker are minimum and maximum for each group. Distribution of the nonsynonymous (**A**) and synonymous (**B**) substitution rate of organellar gene groups across six Caryophyllales taxa. Boxes show the 25% and75% quartiles of the data points, and the horizontal lines within the boxes indicate the median. Whisker lines encompass the range of all non-outlier data points. Points outside the whisker are minimum and maximum for each group. For plastidial gene groups, asterisks above and below the median line denote the significance level of the corresponding gene groups compared to the Rps and Rpl gene groups, respectively. For mitochondrial gene groups, asterisks denote the significance level of the corresponding gene groups compared to the Rp (ribosomal protein) gene group. *, ** and *** denote the significant difference with *P* value less than 0.05, 0.01 and 0.001 in turn under Mann-Whitney test

The structural and mutational dynamics of Caryophyllales plastomes and mitogenomes reveal contrasting trends. While plastomes exhibit a more conserved structural organization and mitogenomes display active structural rearrangements, the mutation rates at nucleotide sites show an inverse pattern. Specifically, the rates of synonymous substitutions (*d*_*S*_), nonsynonymous substitutions (*d*_*N*_), and intron substitutions (*d*) in plastid CDSs and introns are 5.5, 1.6, and 2.3 times higher, respectively, compared to their mitochondrial counterparts (Fig. 6A-C). Notably, introns within tRNA genes are more conserved than those in protein-coding genes of Caryophyllales plastomes, showing a 33% reduction in substitution rate.

In Caryophyllales plastomes, the *d*_*N*_ values for photosynthetic complex genes (mean ~ 0.11–0.12 substitutions/site) are 1.8–5.2 times lower than those for ribosomal protein-coding genes (mean ~ 0.34–0.68 substitutions/site). A similar trend is observed in Caryophyllales mitogenomes for the first time, where the *d*_*N*_ values of oxidative phosphorylation complex genes are 1.1–3.9 times lower than those of ribosomal protein-coding genes (Fig. 6D and E).

**Fig. 7:**
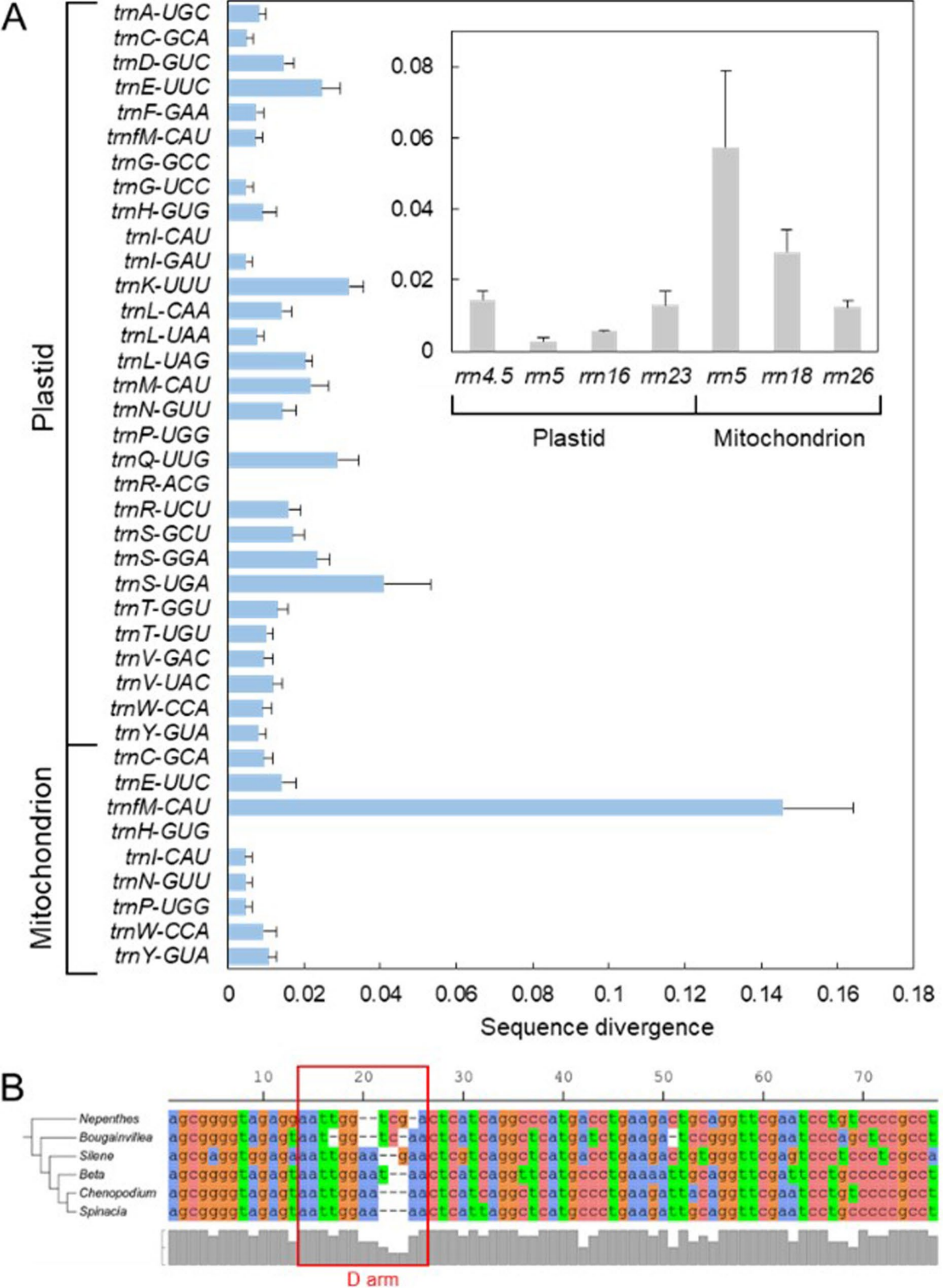
Variation in sequence divergence among organellar tRNA and rRNA genes (**A**) and alignment of *trnfM*-CAT sequences among Caryophyllales taxa (**B**). The columns in chart A show the mean substitution rate of pairwise comparisons among six Caryophyllale taxa and error bars denote the standard errors

The sequence divergence in organellar rRNA and tRNA genes is generally low, at less than 0.04 substitutions/site, with no statistically significant differences between plastidial and mitochondrial genes (Fig. 7). However, the mitochondrial *rrn5* gene exhibits the highest substitution rate among organellar rRNA genes (0.057 substitutions/site), consistent with previously reported elevated mutation rates in the *Silene* genus [11]. Among organellar tRNA genes, the mitochondrial *trnfM*-CAU gene shows an exceptionally high substitution rate (0.15 substitutions/site), approximately twice that of the second most divergent gene, the plastidial *trnS*-UGA gene (Fig. 7B).

## Discussion

The size of the *B*. *glabra* plastome aligns with the recently reported range for the genus (153.9 kb–154.9 kb) [28, 29]. In this study, the mitochondrial genome of Bougainvillea glabra was assembled to a size of several hundred kilobases (kb), placing it at an intermediate scale compared to the known size range of land plant mitogenomes (66 kb–17 Mb) [3]. Unlike most Caryophyllales and other angiosperms, which often harbor large repeats (≥ 1 kb) with variable lengths and sequences [1, 30, 31], the *B*. *glabra* mitogenome lacks large repeats, making it a unique case. Repeats shorter than 1 kb account for 2.9% of the *B*. *glabra* plastome—a typical proportion for Caryophyllales plastomes—whereas their representation in the mitogenome is dramatically higher, at 7.1 times that of the plastome, a phenomenon consistent with other Caryophyllales taxa (Table 1).

The medium repeats in mitochondrial genomes exhibit varying recombination frequencies across taxa. For example, they show extremely high recombination frequencies in the 565 kb *Viscum* mitogenome [32], moderate frequencies in the mitogenomes of *Silene vulgaris*, *Vigna angularis*, and *Mimulus guttatus* (< 530 kb) [33–35], and silent recombination statuses in the larger mitogenomes of *Silene noctiflora*, *Silene conica*, and *Cucumis sativus* (> 1 Mb) [35, 36].

The species of mitochondrial plastid-derived tRNA and ribosomal protein-coding gene groups are mostly changeable in Caryophyllales organellar gene groups (Fig. 3). The changeableness of them may result from the high frequency of endosymbiotic transfer just in different directions that the plastidial tRNA sequences are transferred to mitogenome from time to time at different evolutionary taxa and the transferred tRNA sequences further suffer from elimination pressure that only parts of them can form novel functional genes [37]. In contrast, the mitochondrial ribosomal protein-coding suffer from the functional substitution pressure from the mitochondrion-derived ribosomal protein-coding genes in nuclear genomes [38].

Gene clusters in mitogenomes appear to form at distinct evolutionary stages. For example, the *trnC*-*trnN*-*trnY*, *atp8*-*cox3*, *rps4*-*nad6*, *ccmB*-*trnK*, and *trnH*-*trnE* clusters are distributed among eudicot taxa [39], while *trnY*-*nad2e3-e5*, *rps13*-*nad1*, *nad4L*-*atp4*, *cob*-*rpl5*, and *trnS*-*trnF*-*trnP* clusters are widespread across seed plants [39]. The *rrn18*-*rrn5* cluster can be traced back to the bacterial ancestors of mitochondria [40]. A newly identified *ccmFn*-*ccmFc* cluster in Caryophyllales mitogenomes may date back to a common ancestor approximately 85 million years ago (Ma) (Fig. S4). The transcriptional needs of these clusters likely impose constraints on their evolution, as evidenced by cotranscription events observed in amplified transcripts of gene clusters like *nad4L*-*atp4* and *rps10*-*cox1* in *Nicotiana* and *rpl5*-*rps14*-*cob* in pea and *Arabidopsis* [41–43]. However, the intergenic regions between linked genes exhibit high variability in length and evolutionary rates, distinguishing them from introns and other gene-associated components (Figs. S3, 6).

DNA turnover rates, which represent the loss of homologous DNA over time, highlight the unique evolutionary dynamics of Caryophyllales mitogenomes. These genomes exhibit significantly divergent turnover rates when compared to seed plants [13, 44]. For instance, the turnover rate between Chenopodium and Spinacia (37.4 Ma; ~50% shared DNA) is twice as low as that between *Vigna radiata* and *Glycine max* (19 Ma; ~50% shared DNA), while the rate between *Nepenthes* and five core Caryophyllales taxa (85 Ma; ~20% shared DNA) is 1.5 times higher than that between *Liriodendron tulipifera* and Phoenix (133 Ma; ~20% shared DNA). This trend is supported by the lower correlation coefficient (R² = 0.41) between shared mitochondrial DNA and divergence time in Caryophyllales compared to seed plants (R² = 0.74) [13]. In contrast, DNA turnover in Caryophyllales plastomes shows less divergence, with an R² of 0.56 (Fig. S4A).

MTPTs also vary significantly across Caryophyllales taxa. *Nepenthes ventricosa*, with its highly divergent mitogenome, contains ~ 29.5 kb of plastidial DNA—an exceptionally high amount among seed plants [45]. Other Caryophyllales taxa exhibit lower MTPT content, ranging from 2 to 3 kb in *B*. *glabra* and *Silene* to 6–8 kb in other lineages. In addition to MTPTs, novel transcriptional regions such as *BVmt2_4*, *BVmt2_21*, and *BVmt3_14* (discussed in "Category of organellar transcripts of Bougainvillea glabra" section) may originate from horizontal gene transfer events.

Elimination events, including pseudogenization and intron loss, contribute to detectable DNA turnover, involving approximately 15 kb of sequence loss in Caryophyllales mitogenomes—seven times that observed in plastomes. However, a significant portion of intergenic DNA in organelles remains of unknown origin. Nuclear genomes have been proposed as potential sources of exogenous DNA for plant mitogenomes, pending further sequencing of nuclear genomes in related taxa [1, 37].

In Caryophyllales mitogenomes, the *d*_*N*_ values for oxidative phosphorylation complex genes are consistently lower than those for ribosomal protein-coding genes (Fig. 6D and E). This pattern likely reflects the reduced functional importance of mitochondrial ribosomal protein-coding genes, which can be replaced by homologs from other organelles [1, 38, 46]. Additionally, RNA editing—a key post-transcriptional modification—may also contribute, as nonsilent RNA editing sites exhibit high editing efficiencies and play essential roles in biological processes [25–27].

## Conclusions

We report the first complete *de novo* mitochondrial genome, along with a newly assembled plastid genome and strand-specific organellar transcriptome for *B.glabra*. The *B*. *glabra* mitochondrial genome consists of three autonomous, circular-mapping chromosomes measuring 160.7 kb, 97.6 kb, and 54.3 kb, with a total size of 322.7 kb. Notably, while no large repeats (≥ 1 kb) were identified in the *B*. *glabra* mitogenome, the proportion of repeats shorter than 1 kb is substantially higher (20.6%) than in the plastome (2.9%). Distinct differences between the mitogenome and plastome were also observed in base composition (G + C content: 44.1% vs. 36.4%), gene content (55 vs. 114), and gene density. Transcriptome analysis revealed widespread transcriptional activity in non-coding regions of both organellar genomes. RNA editing analysis identified 43 editing sites in plastidial transcripts and 453 in mitochondrial transcripts, with approximately 70% of these edits occurring at nonsilent sites within coding regions, often with high editing efficiency. A comparative analysis among *B*. *glabra* and five other Caryophyllales taxa demonstrated that the DNA turnover rate in Caryophyllales mitogenomes is 6.1 times faster than in their plastomes. Conversely, nucleotide substitution rates in plastid protein-coding genes are significantly higher than in mitochondrial genes. Substitutions at nonsilent sites of electron transfer chain coding genes were notably constrained compared to those in ribosomal protein-coding genes in both organellar genomes. These findings provide valuable insights into the evolutionary dynamics of *B*. *glabra* and closely related species, offering a foundation for further studies on organellar genome evolution and functional adaptation in Caryophyllales.

## Methods

### Genome sequencing and assembly

Young leaves of *Bougainvillea glabra var*. Formosa were collected from Hainan Botanical Garden of Tropical Economic Plants, Danzhou, Hainan, China. The PacBio library of total leaf DNAs was built following the protocol of SMRTbell Template Prep Kit (Pacific Biosciences, Menlo Park, CA, USA) and sequenced on PacBio sequel (Pacific Biosciences). The sequencing reads of organellar DNAs was extracted using minimap2 version 2.14 (https://github.com/lh3/minimap2) [47] with a -P selection. The reference genomes were chosen from that of the Caryophyllales taxa released in NCBI Genome (https://www.ncbi.nlm.nih.gov/datasets/organelle/) by Sep. 2019 and an *B. buttiana* mitogenome obtained through experimental extraction of mitochondrial DNAs and Illumina sequencing. A total of 294,316 and 112,175 reads were filtered as plastidial and mitochondrial origin, respectively. The length distribution of these PacBio reads was shown in Figs. S1, A and B. The extracted organellar reads were *de*-*novo* assembled through a guidance-base assembly approach using OrganellarRef_PBA (https://github.com/aubombarely/Organellar_PBA) [48]. The repetitive assembly at the scaffold ends was discarded and the final assemblies were confirmed by manual detection for the continuity of PacBio mapping reads in Tablet version 1.19 (https://ics.hutton.ac.uk/tablet) [49].

### Genome annotation and repeat identification

The assembled organellar genomes of *B*. *glabra* were annotated using GeSeq (https://chlorobox.mpimp-golm.mpg.de/geseq.html) [50] with the reference organellar genomes of *Beta vulgaris* (KR230391.1, NC_015099.1), *Chenopodium quinoa* (NC_034949.1, NC_041093.1), *Spinacia oleracea* (NC_002202.1, NC_035618.1), *Silene latifolia* (NC_016730.1, NC_014487.1) and *Nepenthes ventricosa* (NC_041271.1, NC_039531.1). The annotations were further checked manually to avoid the loss of gene components. Novel mitochondrial transcripts were annotated after the identification of organellar transcripts described in part 5.6. The circular organellar map was drawn using OGDRAW version 1.1 (https://chlorobox.mpimp-golm.mpg.de/OGDraw.html) [51]. Repeats larger than 19 bp were identified using NCBI-BLASTN version 2.8 with -word_size 7 and -min_raw_gapped_score 19. Tandem repeats with a unit size larger than six were identified using TRF version 4.09 (https://tandem.bu.edu/trf/trf.html) under default settings, and other tiny tandem repeats were analyzed using MISA version 1.0 (http://misaweb.ipk-gatersleben.de/) [52]with the definition (unit_size, min_repeats): 1–8, 2–4, 3–4, 4 − 3, 5 − 3 and 6 − 3. The total length of different types of repeats was calculated in the non-redundancy genomes excluding the redundant copies of large repeats using an in-house pipeline.

### Comparative analysis of organellar genomes

Five Caryophyllales species with both organellar genomes were selected from NCBI-genome, including the genomes of *Beta vulgaris* (KR230391.1, NC_015099.1), *Chenopodium quinoa* (NC_034949.1, NC_041093.1), *Spinacia oleracea* (NC_002202.1, NC_035618.1), *Silene latifolia* (NC_016730.1, NC_014487.1) and *Nepenthes ventricosa* (NC_041271.1, NC_039531.1). Multiple comparisons of the organellar genomes of *B*. *glabra* and these Caryophyllales taxa were performed using BLASTN [53] with relax parameters: -perc_identity 65 after masking the repeats larger than 1 kb as a single copy. Synteny regions were integrated in BEDTools version 2.25 (https://bedtools.readthedocs.io/en/latest) [54] under the merge command. The turnover rate was calculated by dividing the shared DNAs by the divergence time of any two taxa recorded on Timetree web (http://www.timetree.org/) [55]. Transferred DNAs were identified from the homologous sequences of different original genomes by judging their conservative degree in different genomes. The conservative sequences of protein-coding and rRNA genes of plastomes and mitogenomes were masked before DNA transfer analysis to avoid ambiguous judgment.

### Transcriptome sequencing

Leaf RNAs were isolated using TRIzol (Invitrogen, Carlsbad, USA) and the ribosomal RNAs were depleted from the RNA sample through hybridization to the complementary oligonucleotides using Ribo-Zero rRNA Removal Kit (Epicentre, Madison, WI, USA). A strand-specific RNA library was generated using the NEBNext Ultra Directional RNA Library Prep Kit for Illumina (NEB, Ipswich, MA, USA) following the manufacturer’s recommendations. The clustering of the index-coded sample was performed on a cBot Cluster Generation System using TruSeq PE Cluster Kit v4-cBot-HS for Illumina (NEB, Ipswich, MA, USA) according to the manufacturer’s instructions. After cluster generation, the library was sequenced at Biomarker Technologies Co., Ltd (Beijing, China) on the Hiseq Xten platform (Illumina, San Diego, CA, USA), which totally generated 57.1 million 2 × 150 bp reads (SRA accession number: SRR10082746).

### Transcriptome assembly

Raw data were processed in SOAPnuke version 1.5 (https://github.com/BGI-flexlab/SOAPnuke) [56] to remove the adapters of reads, reads containing more than 5% poly-N sequences and low-quality reads in which at least 50% of bases showed quality of less than 15. The trimmed reads were mapped to *B*. *glabra* organellar genomes using TopHat version 2.1 (ccb.jhu.edu/software/tophat/index.shtml) [57] with relaxed parameters: -N 4, -read-gap-length 0, -read-edit-dist 4, -max-insertion-length 0 and -max-deletion-length 0. To avoid the mismatch of the reads of plastidial transcript to the MTPTs or paralogous mitochondrial genes, we combined the plastidial and mitochondrial genomes together as the reference genome. The reads in the same orientation were classified using an in-house shell script, respectively. The coverage depth of transcriptomic data on each stand of organellar genomes was stated using samtools version 1.0 (samtools.sourceforge.net) [58], respectively.

### Novel transcript identification

The organellar transcripts of *B*. *glabra* were assembled using StringTie version 1.3 (ccb.jhu.edu/software/stringtie) [59] with relaxed parameters: -m 30 and -c 3. The assembled transcripts were compared with the annotations of *B*. *glabra* organellar genomes using Cuffcompare version 2.2 (cole-trapnell-lab.github.io/cufflinks/cuffcompare) [57]. Novel organellar transcripts were screened out when satisfying conditions of FPKM ≥ 0.5, coverage ≥ 3, and class code with u, i and x. The coding or noncoding transcripts (cRNAs or ncRNAs) were recognized using Coding Potential Calculator (CPC, http://cpc2.cbi.pku.edu.cn/) [60]. The long noncoding RNAs (lncRNAs) were identified from ncRNAs with length > 200 bp and ORF < 120 amino acid (AA) using UGENE version 1.32 (ugene.net) [61]. The expression level of organellar transcripts was calculated using StringTie [59].

### RNA editing analysis

RNA editing sites of organellar transcripts were identified through a left tail Fisher test for the distribution of expected/observed bases at all the covered genomic positions using REDItoolDenovo.py script in REDItools version 1.2 (reditools.sourceforge.net) [62] with moderate parameters: -V 0.05, -c 10, -n 0.1 and -r 4. The editing sites of the genes on different strands of the organellar chromosomes were identified, respectively. The effect of RNA editing on organellar transcripts was predicted using SnpEff version 4.3t (snpeff.sourceforge.net) [63] and the interval length of the upstream or downstream of genes was set as 1 kb.

### Substitution rate estimation

The coding regions and introns of the protein-coding genes, tRNA genes and rRNA genes shared in Caryophyllales organellar genome were selected for substitution rate analyses. Sequence alignment of each gene was performed in MAFFT version 7.313 (https://mafft.cbrc.jp/alignment/software) [64] using default parameters. Poorly aligned regions were eliminated using GBLOCKS version 0.91b (http://molevol.cmima.csic.es/castresana/Gblocks.html) [65] with the following settings: b1 = h + 1, b2 = h + 1, b4 = 5 and b5 = h. Numbers of synonymous and non-synonymous substitutions per site (*d*_*S*_ and *d*_*N*_) of protein-coding genes were calculated using the maximum likelihood method within the codeml application in PAML version 4.9 h (web.mit.edu/6.891/www/lab/paml.html) [66] with following options: seqtype = 1, runmode = −2 and CodonFreq = 2. Substitution rate (*d*) of non-coding regions was estimated using baseml application with HKY85 substitution model for introns, *rrn18* and *rrn26* and K80 model for *rrn5* and tRNA genes. RNA editing sites were not excluded in the present analyses because empirical editing data were not available for all of these taxa.

## Supplementary Information

Supplementary Material 1.

Supplementary Material 2.

## Data Availability

For RNA-seq data, we used leaves sample data of B. glabra ‘Forsoma’ in NCBI Sequence Reads Archive (SRA) under the accession number SRR10082746. The datasets supporting the conclusions of this article are included in the article and its additional files.

## Abbreviations

Mitogenomes: mitochondrial genomes
plastomes: plastid genomes
MTPTs: Mitochondrial plastid transferred fragments
lncRNAs: long noncoding RNAs
AA: amino acid
cRNAs or ncRNAs: coding or noncoding transcripts
TRs: tandem repeats
CV: coefficient of variation
CDS: coding sequence
ORFs: open reading frames
