# Supplementary material for: Comparative genomics uncovers organellar genome structural divergence in Caryophyllales and reveals widespread non-coding transcription in Bougainvillea glabra organellar
Source: BMC Genomics. 2025 Aug 4;26:722. doi: 10.1186/s12864-025-11891-5 (PMC12323158; doi:10.1186/s12864-025-11891-5)
Supplement: Supplementary file 2 — Supplementary Material 2. [file 12864_2025_11891_MOESM2_ESM.docx]

**Supplementary Figures**

Characterization of the complete organellar genomes of *Bougainvillea glabra* and the genomic comparisons among Caryophyllales

Shuo Zhang ^a, b^ †, Shengxin Chang ^a, c^ †, Xinge Lin ^a^ †, Shisong Xu ^a^, Qingyun Leng ^a^, Haiyan Li ^a^, Hernán Ariel López ^d^, Zhiqiang Wu ^a^ *, and Junhai Niu ^a^ *


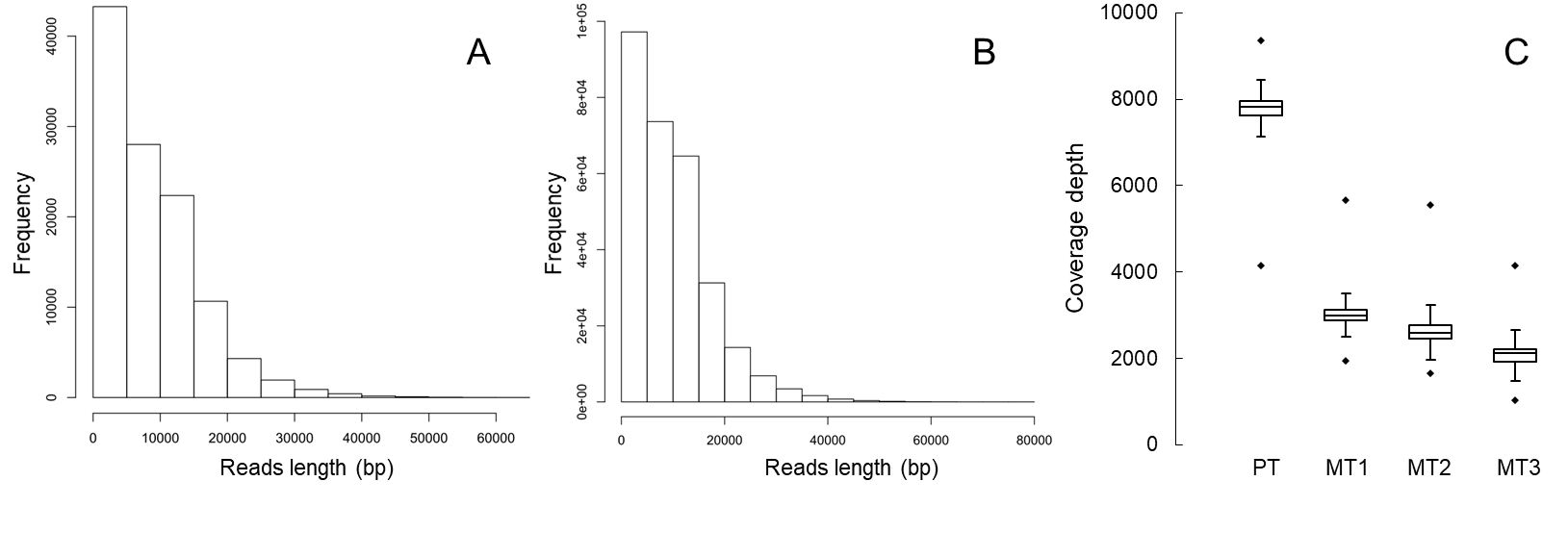


**Supplementary Fig.1.** Length distribution of the PacBio reads of plastidial and mitochondrial DNAs (A and B) and reads coverage depth on each organellar chromosome (C).


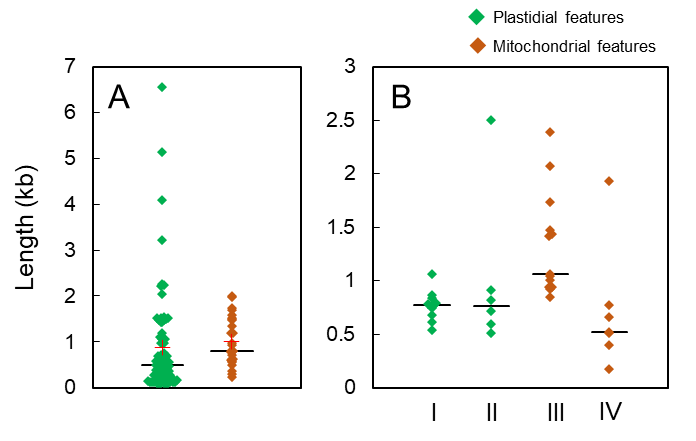


**Supplementary Fig.2.** Length distribution of organellar CDSs (A), introns and intergenic regions of mitochondrial gene clusters (B) in Caryophyllales. I - introns of plastidial protein-coding genes. II - introns of plastidial tRNAs. III - introns of mitochondrial introns. IV - intergenic regions of mitochondrial gene clusters. Each point in A and B charts denotes the average length of a gene feather. Black horizontal lines and red crosses indicate the medians and means of each group, respectively.

| 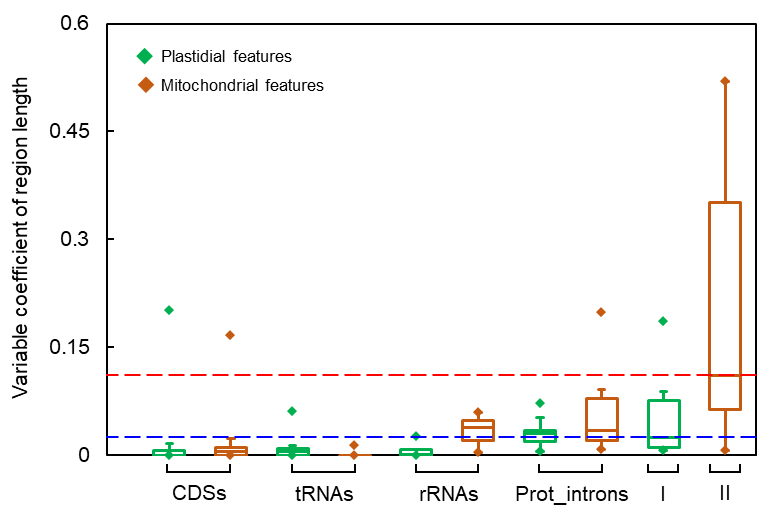 |
| --- |
| **Supplementary Fig.3.** Variable coefficient of the length of organellar gene features. Prot_introns – introns of protein coding genes. I – introns of plastidial tRNAs. II – intergenic regions of mitochondrial gene clusters. Boxes show the 25% and75% quartiles of the data points, and horizontal lines within the boxes indicate the medians. Whisker lines encompass the range of all non-outlier data points. Points in black indicate the minimum and maximum of each group. |


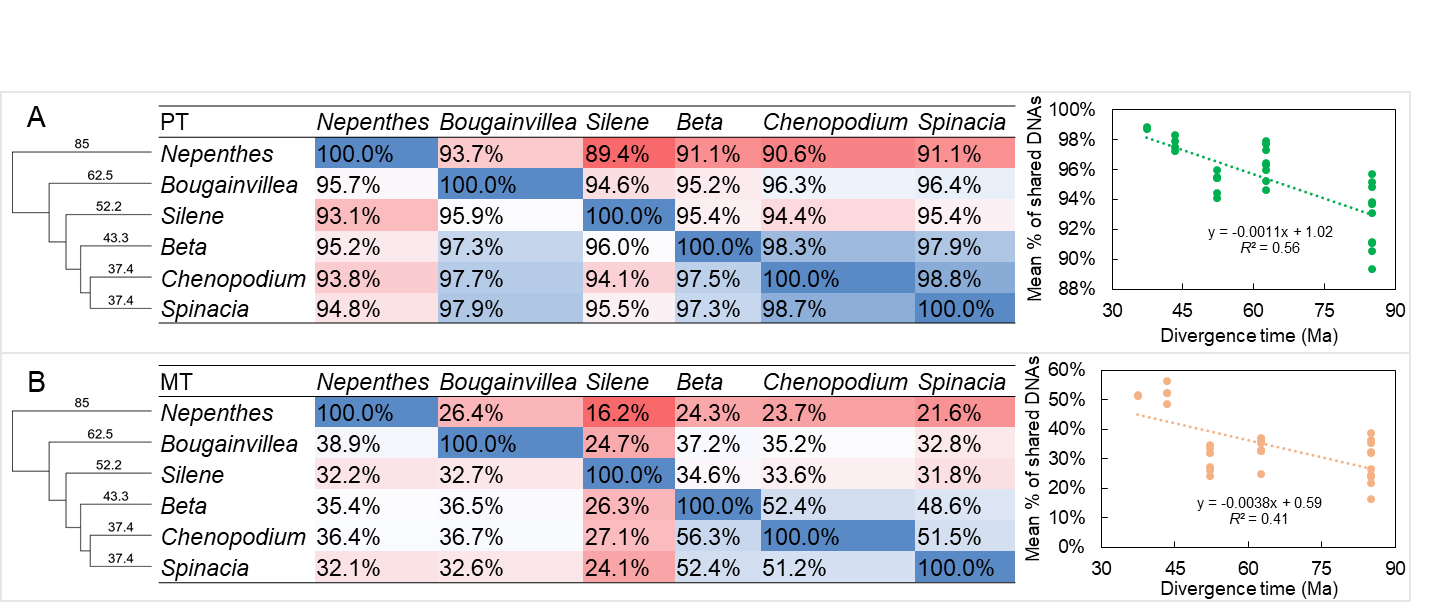


**Supplementary Fig.4.** Proportion matrix of shared DNAs of Caryophyllales plastomes (A) and mitogenomes (B). The redundant large repeats were removed before analyses. The left tree gives the phylogenetic relationship and divergence time (Ma) of six Caryophyllales taxa according to APW (http://www.mobot.org/mobot/research/apweb/) and Timetree web (http://www.timetree.org/). The right pictures show the pairwise proportion of shared DNAs as a function of divergence time.

| 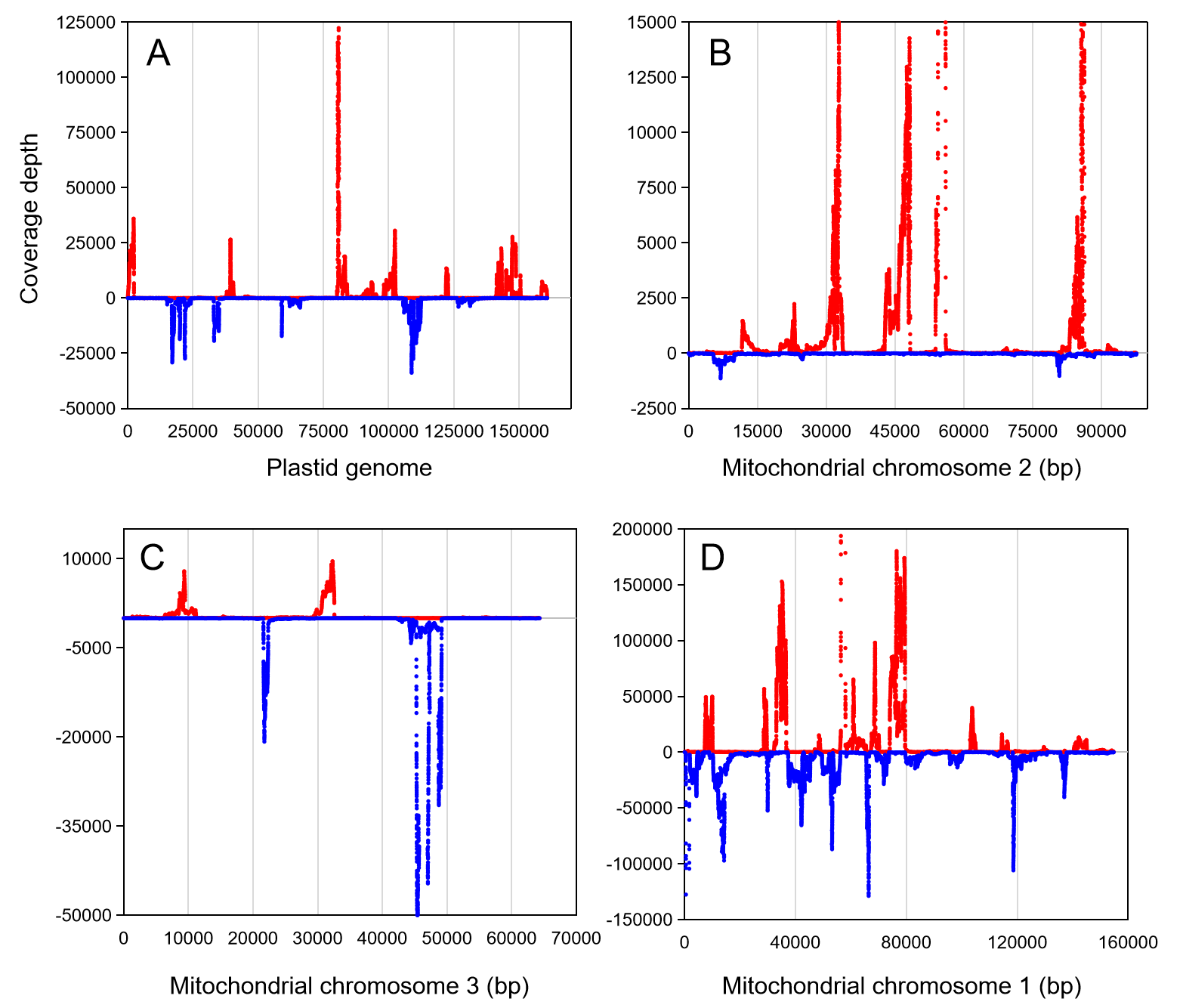 |
| --- |
| **Supplementary Fig.5.** Directional mapping of the transcriptomic reads of *B. glabra* on its organellar chromosomes*.* Red and blue colors indicate the opposite directions of the sequencing reads. |


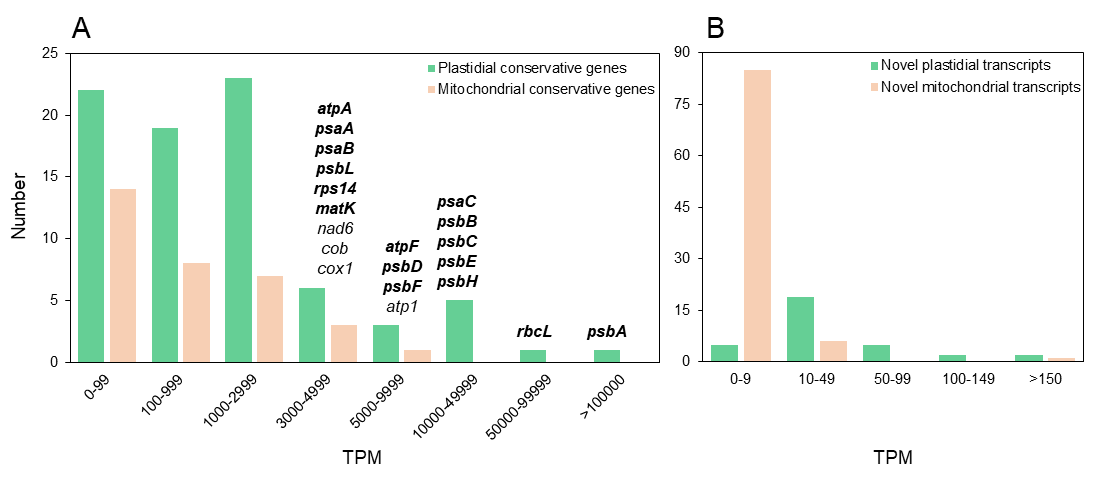


**Supplementary Fig.6.** Histogram of transcripts per million reads (TPM) of conservative organellar protein-coding genes (A) and predicted organellar transcripts (B).

| 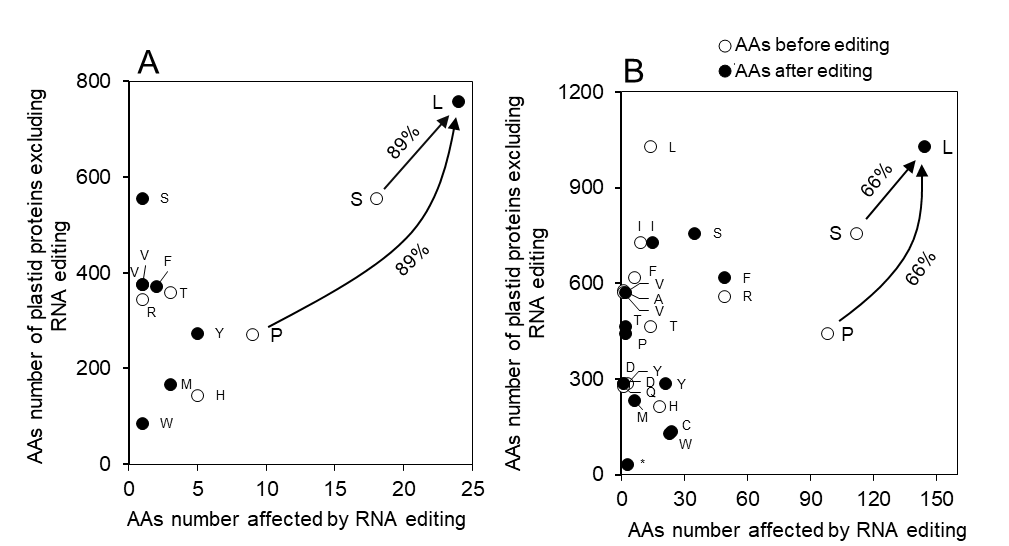 |
| --- |
| **Supplementary Fig.7.** Number of codons encoding amino acids (AAs) affected by RNA editing in *Bougainvillea* plastome (A) and mitogenome (B). Y axes denote the total number of AAs in organellar proteins. X axes denote the total number of AAs affected by RNA editing. Solid circles represent the AA codons before RNA editing, while hollow circles represent the AA codons after RNA editing. L-leucine. S-serine. P-proline. |
